# Safety and adherence of bictegravir/emtricitabine/tenofovir alafenamide for HIV post-exposure prophylaxis among adults in Guiyang China: a prospective cohort study

**DOI:** 10.1186/s12879-024-09407-9

**Published:** 2024-06-06

**Authors:** Lin Gan, Xiaoxin Xie, Yanhua Fu, Xiaoyan Yang, Shujing Ma, Linghong Kong, Chunli Song, Yebing Song, Tingting Ren, Hai Long

**Affiliations:** 1 Guiyang Public Health Clinical Center, 6 Daying Road, Yunyan District, Guiyang, 550001 China; 2 School of Public Health, The Key Laboratory of Environmental Pollution Monitoring and Disease Control, Ministry of Education, Guizhou Medical University, Guiyang, 550025 China

**Keywords:** HIV post-exposure prophylaxis, Bictegravir/emtricitabine/tenofovir alafenamide, Single-tablet regimen, Integrase strand transfer inhibitors, Safety

## Abstract

**Background:**

The effectiveness of post-exposure prophylaxis (PEP) depends on participants adherence, making it crucial to assess and compare regimen options to enhance human immunodeficiency virus (HIV) prophylaxis strategies. However, no prospective study in China has shown that the completion rate and adherence of single-tablet regimens in HIV PEP are higher than those of multi-tablet preparations. Therefore, this study aimed to assess the completion rate and adherence of two HIV PEP regimens.

**Methods:**

In this single-center, prospective, open-label cohort study, we included 179 participants from May 2022 to March 2023 and analyzed the differences in the 28-day medication completion rate, adherence, safety, tolerance, and effectiveness of bictegravir/emtricitabine/tenofovir alafenamide (BIC/FTC/TAF) and tenofovir disoproxil fumarate, emtricitabine, and dolutegravir (TDF/FTC + DTG).

**Results:**

The PEP completion rate and adherence were higher in the BIC/FTC/TAF group than in the TDF/FTC + DTG group (completion rate: 97.8% vs. 82.6%, *P* = 0.009; adherence: 99.6 ± 2.82% vs. 90.2 ± 25.29%, *P* = 0.003). The incidence of adverse reactions in the BIC/FTC/TAF and TDF/FTC + DTG groups was 15.2% and 10.3% (*P* = 0.33), respectively. In the TDF/FTC + DTG group, one participant stopped PEP owing to adverse reactions (1.1%). No other participants stopped PEP due to adverse events.

**Conclusions:**

BIC/FTC/TAF and TDF/FTC + DTG have good safety and tolerance as PEP regimens. BIC/FTC/TAF has a higher completion rate and increased adherence, thus, is recommended as a PEP regimen. These findings emphasize the importance of regimen choice in optimizing PEP outcomes.

**Trial registration:**

The study was registered in the Chinese Clinical Trial Registry (registration number: ChiCTR2200059994(2022-05-14), https://www.chictr.org.cn/bin/project/edit?pid=167391).

## Background

The Joint United Nations Programme on human immunodeficiency virus/acquired immunodeficiency syndrome (HIV/AIDS) (UNAIDS) reported that there were 39 million people living with HIV/AIDS and 1.3 million new infections worldwide in 2022 [1]. The epidemic forecast is not optimistic, and the government of China has adopted a series of measures to control the spread of HIV. In 2019, the Notice on Printing and Distributing the Implementation Plan to Stop the Spread of AIDS (2019–2022) was issued, emphasizing the importance of post-exposure prophylaxis (PEP) [2].

PEP, a biological means to block the spread of HIV [3], usually comprises three antiretroviral drugs that are used continuously for 28 days, starting within 72 h after exposure [4]. Although no randomized controlled trial has reported on the effectiveness of PEP in humans, its efficacy was confirmed in non-human primate models in the early 1990s [5, 6] and was subsequently shown to be effective in humans in a case-control study in 1997 [7]. Many subsequent observational studies have confirmed the effectiveness of PEP [8–18].

Usually, a regimen composed of three antiretroviral drugs is recommended for PEP [19]; however, the early PEP regimen is not well-tolerated, which led to a low completion rate [20]. With the wide clinical application of integrase inhibitors, their high efficiency and low toxicity make them potential candidates for PEP [21]. According to China’s guidelines [22], a single-tablet regimen (STR) consisting of bictegravir, emtricitabine, and tenofovir alafenamide (BIC/FTC/TAF) and the multi-tablet regimen (MTR) consisting of tenofovir disoproxil fumarate, emtricitabine, and dolutegravir (TDF/FTC + DTG) are the first choice for PEP.

Poor adherence and low completion rates are the main factors affecting the effectiveness of PEP [14, 15], and studies have shown that individuals treated with the STR have a higher completion rate and better adherence than those treated with the MTR [8, 15, 17]. However, to the best of our knowledge, no prospective study conducted in China has confirmed this finding. At present, the most frequently used PEP regimens in China are TDF/FTC + DTG and BIC/FTC/TAF, therefore, we designed a prospective cohort study to compare the completion rate and level of adherence to BIC/FTC/TAF with those of TDF/FTC + DTG for PEP and explore the safety and tolerance of these two regimens for PEP.

## Methods

### Study design and participants

This single-center, prospective, open-label cohort study was conducted at the Guiyang Public Health Clinical Center, one of the largest infectious disease hospitals in Southwest China, between May 2022 and March 2023. The participants chose BIC/FTC/TAF or TDF/FTC + DTG according to their preference, and the follow-up lasted 12 weeks. Participants’ choice of regimen was based on Chinese guidelines [22] or cost (the cost of BIC/FTC/TAF was about 1100 ¥, and the cost of TDF/FTC + DTG was about 2800 ¥).

All participants were required to provide an exposure history, and the first dose of drugs was administered within 72 h of evaluation by the attending physician. All participants underwent rapid HIV antibody detection, and the inclusion criteria were as follows: (1) age > 18 years, regardless of sex; (2) no infection with HIV; (3) exposure to HIV within 72 h, including but not limited to unprotected sex (anal, vaginal, oral, etc.) with people who are HIV-positive (with an unknown or detectable viral load) or whose infection status is unknown, and damaged skin or mucous membranes coming into contact with body fluids such as blood, semen, and vaginal secretions of people suspected or confirmed to have HIV (with an unknown or detectable viral load); (4) fertile women willing to take contraceptive measures during the study drug taking period; and (5) provision of signed informed consent. The exclusion criteria were as follows: (1) exposure time exceeding 72 h, (2) intolerance or allergy to drugs or auxiliary materials used in the study; and (3) chronic/active hepatitis B (HBV).

The main end point of this study was the proportion of participants who completed PEP for 28 days (PEP completion rate). For the participants who did not visit the outpatient clinic for follow-up after 28 days of PEP, a member of the research team called the participant to check whether they had completed the medication and asked about their tolerance of the drugs. The secondary endpoints included adherence and HIV infection rates at weeks 4 and 12.

### Procedures

After the participants provided informed consent and were enrolled in the study, a nurse recorded their demographic information and exposure details, and the attending physician advised them to take the first dose of drugs as soon as possible and then conducted blood tests, including routine blood tests, blood biochemistry, urine analysis, and assessment of HBV markers, hepatitis C antibody, and syphilis markers. Routine follow-ups were scheduled at days 14 and 28, and weeks 8 and 12. During each follow-up, blood, urine, HIV antibody, HBV markers, hepatitis C antibody, and syphilis markers were examined. Adherence (through drug dose evaluation: actual dose/28 × 100%) and adverse drug reactions were evaluated concurrently.

### Ethics approval and informed consent

The study was registered in the Chinese Clinical Trial Registry (registration number: ChiCTR2200059994 (2022-05-14), https://www.chictr.org.cn/bin/project/edit?pid=167391), was approved by the Ethics Committee of the Guiyang Public Health Clinical Center (202212), and was conducted in accordance with the standards laid out in the Declaration of Helsinki. All participants provided written informed consent.

### Statistical analysis

According to the literature [17], the proportion of participants who complete preventive medication for 28 days can reach 90% in the BIC/FTC/TAF group and 60% in the TDF/FTC + DTG group. We calculated that a sample of 64 patients (32 in the BIC/FTC/TAF group; 32 in the TDF/FTC + DTG group) would provide the study with 90% power to detect a difference between the group proportions of 30% with a one-sided alpha of 0.05. Given an anticipated dropout rate of 20%; therefore, the total sample size required was at least 80 participants (BIC/FTC/TAF group at least 40; TDF/FTC + DTG group at least 40).

Continuous variables were compared using Student’s t-test or the Mann–Whitney U-test, and categorical variables were compared using the χ^2^ or Fisher’s exact test. The Kolmogorov–Smirnov test was used to determine whether continuous variables fit the normality assumption. A multivariable logistic regression model was created to adjust for confounders and identify potential factors associated with failure to complete the 28 days of medication. The covariates included sex, age, mode of exposure, regimen, educational level, and exposure time.

## Results

### Study participants and baseline characteristics

**Fig. 1:**
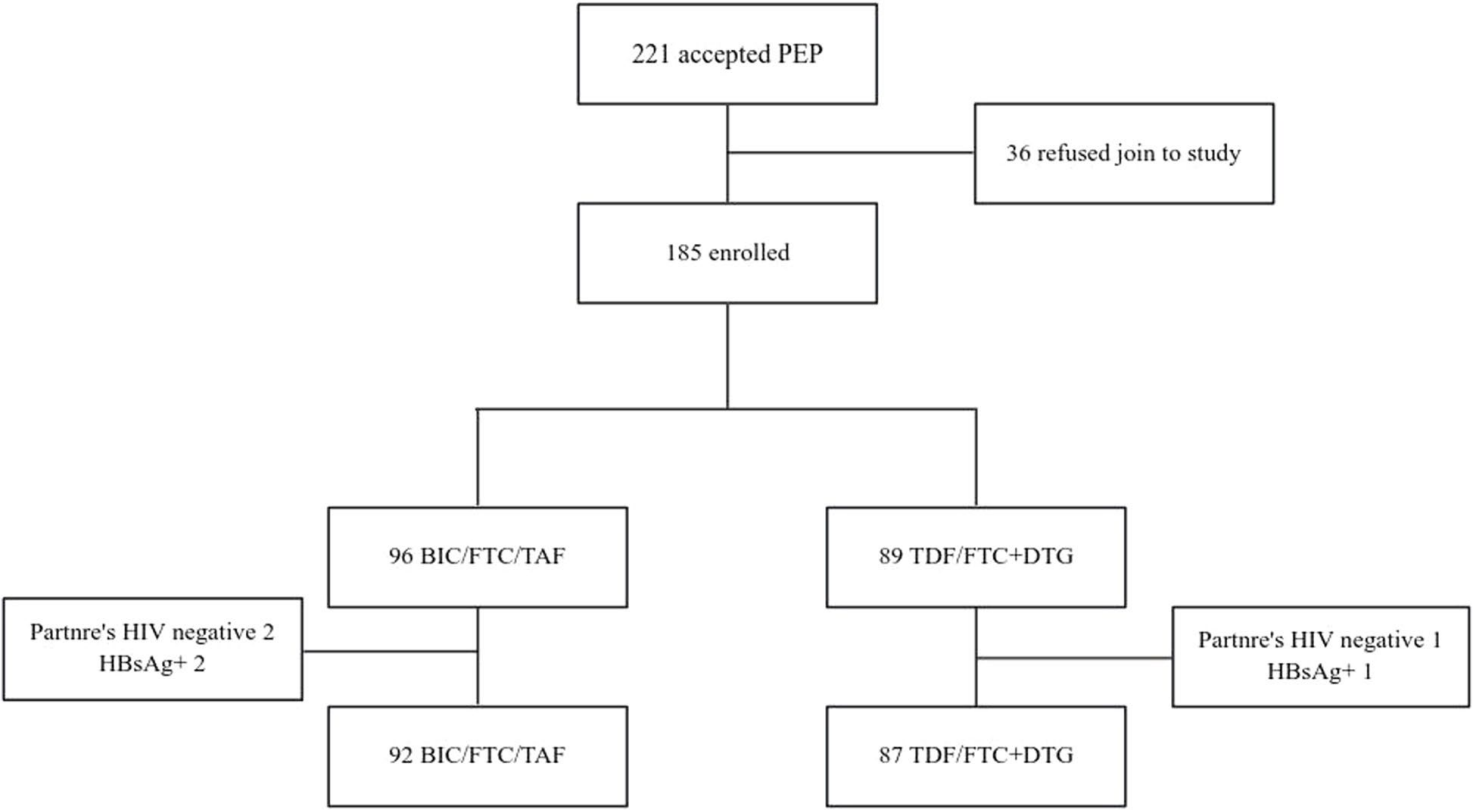
Flowchart showing participant inclusion and attendance to follow-up in the study

The participant selection process is shown in Fig. 1. Between May 2022 and March 2023, a total of 96 received BIC/FTC/TAF, of whom 2 were excluded because they tested HBsAg positive, and another 2 were excluded because the suspected source of exposure was confirmed to be negative. Finally, 92 participants were included in the BIC/FTC/TAF group in the analysis. A total of 89 participants received TDF/FTC + DTG, among whom 1 participant was excluded because they tested HBsAg-positive, and another participant was excluded because their exposure source was confirmed to be negative. Therefore, 87 participants were included in the TDF/FTC + DTG group.

The participants in this study were mainly men (88.3%), with a median age of 29 (interquartile range [IQR]: 25–35) years. The participants in the TDF/FTC + DTG group were older than those in the BIC/FTC/TAF group (31 [26–37] and 27.5 [25–34] years, respectively). Regarding mode of exposure, participants in the BIC/FTC/TAF and TDF/FTC + DTG groups mainly had vaginal intercourse (77.2% and 75.9%, respectively), and the median (IQR) exposure times were 17 (12.0–33.2) h and 17 (12.0–34.0) h, respectively. Overall, 63% and 67.8% started PEP within 24 h of exposure in the BIC/FTC/TAF and TDF/FTC + DTG groups, respectively. Four patients in the BIC/FTC/TAF group and two patients in the TDF/FTC + DTG group had previously received PEP. The patients’ baseline characteristics and differences between the groups are summarized in Table 1.

**Table 1:** Baseline demographic and clinical characteristics

Characteristic	Total (*n* = 179)	BIC/FTC/TAF (*n* = 92)	TDF/FTC + DTG (*n* = 87)	*P* value
Sex, n (%)				0.575
Male	158 (88.3)	80 (86.9)	78 (89.7)	
Female	21 (11.7)	12 (13.1)	9 (10.3)	
Age, median (IQR), year	29 (25–35)	27.5 (25–34)	31 (26–37)	0.042
Mode of exposure, n (%)				0.898
Vaginal intercourse	137 (76.5)	71 (77.2)	66 (75.9)	
Anal sex	35 (19.6)	17(18.5)	18(20.7)	
Oral sex	7 (3.9)	4(4.3)	3(3.4)	
Educational level, n (%)				0.461
Undergraduate degree or above	122 (68.2)	65 (70.7)	57 (65.5)	
Senior high school and below	57 (31.8)	27 (29.3)	30 (34.5)	
Median exposure time, n (%), h	17 (12–34)	17 (12.0–33.2)	17 (12.0–34.0)	0.987
< 24	117 (65.4)	58 (63)	59 (67.8)	
24-47.9	40 (22.3)	26 (28.3)	14 (16.1)	
48–72	22 (12.3)	8 (8.7)	14 (16.1)	
Previous PEP	6 (3.4)	4 (4.3)	2 (2.3)	0.729
Hepatitis C Ab (+), n (%)	1 (0.6)	0 (0)	1 (1.1)	0.486
*Treponema pallidum* Ab (+), n (%)	9 (5)	6 (6.5)	3 (3.4)	0.549

*Ab* Antibody, *BIC/FTC/TAF* Bictegravir emtricitabine and tenofovir alafenamide, *IQR* Interquartile range, *PEP* Post-exposure prophylaxis, *TDF/FTC + DTG* Tenofovir disoproxil fumarate emtricitabine and dolutegravir

### PEP completion rate and compliance

**Fig. 2:**
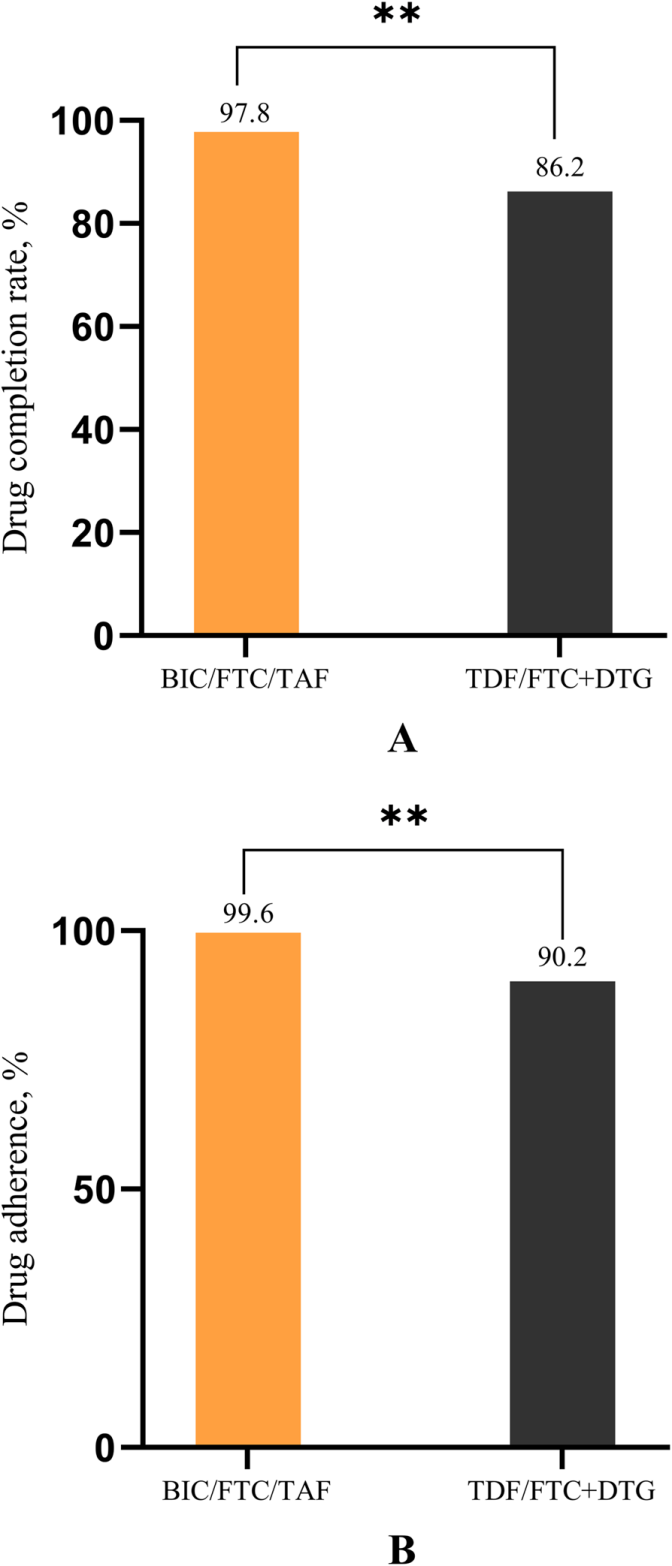
HIV post-exposure prophylaxis (PEP) completion rate and adherence. **a** PEP completion rates in the BIC/FTC/TAF and TDF/FTC + DTG groups. **b** Adherence of the BIC/FTC/TAF and TDF/FTC + DTG groups. Abbreviations: BIC/FTC/TAF: bictegravir, emtricitabine, and tenofovir alafenamide; TDF/FTC + DTG: tenofovir disoproxil fumarate, emtricitabine, and dolutegravir

The PEP completion rate was 97.8% (90/92) in the BIC/FTC/TAF group and 86.2% (75/87) in the TDF/FTC + DTG group (*P* = 0.009) (Fig. 2a). The adherence rate of participants was 99.6 ± 2.82% in the BIC/FTC/TAF group and 90.2 ± 25.29% in the TDF/FTC + DTG group (*P* = 0.003) (Fig. 2b).

A multivariable logistic regression model was created to identify the factors associated with incomplete PEP adherence (Table 2). After adjusting for potential confounders, preventive drug regimen was the only factor associated with incomplete PEP (adjusted odds ratio [aOR] TDF/FTC + DTG vs. BIC/FTC/TAF: 7.02, 95% confidence interval (CI): 1.82–46.29].

**Table 2:** Multivariable regression model for risk factors for not completing PEP

Characteristic	Unadjusted OR (95% CI)	Adjusted aOR (95% CI)
Sex		
Female	1 (ref.)	
Male	0.78 (0.19–5.26)	
Age, per 1-year increase	0.98 (0.91–1.04)	
Mode of exposure		
Anal sex	1 (ref.)	
Oral sex	5.67 (0.21–157.39)	
Vaginal intercourse	3.26 (0.61–60.5)	
Regimen		
BIC/FTC/TAF	1 (ref.)	
TDF/FTC + DTG	7.2 (1.89–47.2)^a^	7.02 (1.82–46.29)^a^
Educational level		
Undergraduate degree or above	1 (ref.)	
Senior high school and below	3.16 (1.04–10.04)^a^	
Exposure time, per 1-hour increase	1.02 (0.99–1.05)	

*Abbreviations:* *aOR* Adjusted odds ratio, *BIC/FTC/TAF* Bictegravir emtricitabine and tenofovir alafenamide, *CI* Confidence interval, *OR* Odds ratio, *ref.* Reference group, *TDF/FTC + DTG* Tenofovir disoproxil fumarate emtricitabine and dolutegravir

^a^*P* < 0.05

### Treatment outcomes

In this 12-month study, no participants were HIV-positive. At the 28-day follow-up, 85.9%(79/92) participants in the BIC/FTC/TAF group and 79.3%(69/87) participants in the TDF/FTC/+DTG group tested negative for HIV antibodies during outpatient visits. The remaining participants reported negative HIV antibody results by telephone interview. At the 12-week follow-up, 67.4%(62/92) participants in the BIC/FTC/TAF group and 69%(60/87) participants in the TDF/FTC/+DTG group tested negative for HIV antibodies during outpatient visits, The remaining participants reported negative HIV antibody results by telephone interview.

### Safety

**Fig. 3:**
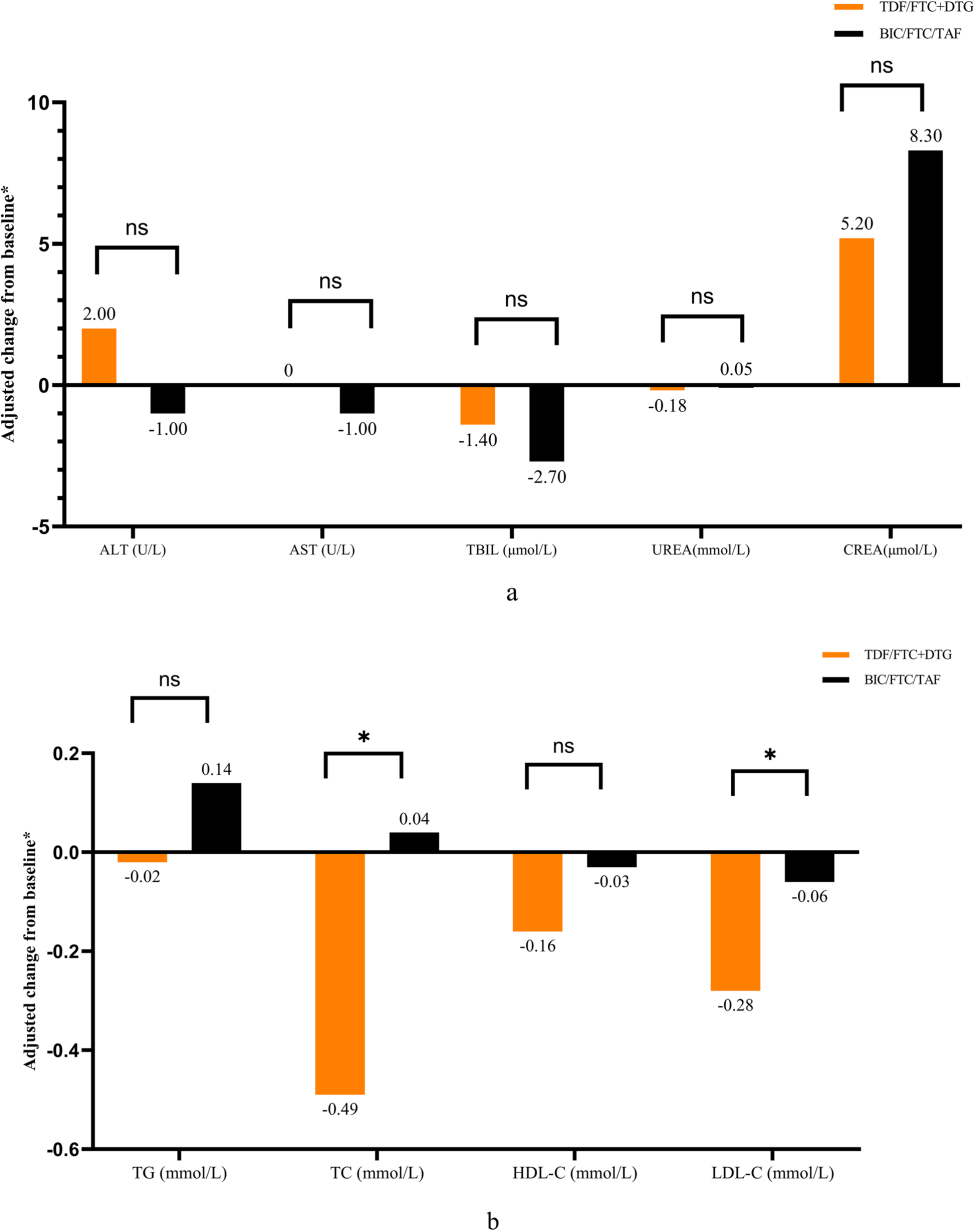
Changes in biomarkers of different PEP regimens from baseline to 28 days

 The overall incidence of adverse drug reactions (ADRs) was 15.2% in the BIC/FTC/TAF group, of which the most common were dyslipidemia (5.4%), hepatic function abnormalities (2.2%), increased blood uric acid levels (2.2%), and elevated serum creatinine levels (2.2%). The overall incidence of ADRs was 10.3% in the TDF/FTC + DTG group, of which the most common were dyslipidemia (3.4%) and increased blood uric acid levels (3.4%) (Fig. 3). One participants in the TDF/FTC + DTG group stopped PEP because of dizziness (Table 3). Among all the participants, the ADRs were grades 1–2.

**Table 3:** Overview of adverse drug reactions (n, %)

Number of grade 1–2 ADRs	BIC/FTC/TAF(*n* = 92)	TDF/FTC + DTG (*n* = 87)	*P* value
**All ADRs**	14 (15.2)	9 (10.3)	0.33
Dyslipidemia	5 (5.4)	3 (3.4)	
Hepatic function abnormal	2 (2.2)	1 (1.1)	
Blood uric acid increased	2 (2.2)	3 (3.4)	
Bilirubin elevation	1 (1.1)	1 (1.1)	
Platelet reduction	1 (1.1)	0 (0)	
Elevated serum creatinine	2 (2.2)	1 (1.1)	
Pyrexia	1 (1.1)	0 (0)	
**Drug-related ADRs**			
Dizziness	0 (0)	1 (1.1)	

*ADR* Adverse drug reaction, *BIC/FTC/TAF* Bictegravir emtricitabine and tenofovir alafenamide, *TDF/FTC + DTG* Tenofovir disoproxil fumarate emtricitabine and dolutegravir

## Discussion

By the end of this prospective cohort study, no participants were HIV-positive, and TDF/FTC + DTG and BIC/FTC/TAF showed good effectiveness, safety, and tolerance. However, this study found that the PEP completion rate of the BIC/FTC/TAF group is significantly higher than that of the TDF/FTC + DTG group. Therefore, we consider that BIC/FTC/TAF should be considered the first choice for PEP.

In our cohort, the participants were mainly young men with a median age of 29 years, which was similar to the findings of other studies in China [12, 13]. The incidence of AIDS among older adults in China has been increasing each year [23]. Because older adults in China have a low level of understanding of diseases and PEP, PEP uptake among older adults is low. This may explain why participants in PEP studies in China, including our study, tend to be young adults.

In this study, vaginal intercourse was the main mode of exposure, which is consistent with another study conducted in Southwest China [13]. However, this was in contrast with two other prospective studies conducted in France and Beijing, in which the main mode of exposure was anal sex, accounting for 64% and 51.8% of exposures, respectively.

Our results show that the PEP completion rate and adherence were higher with the STR than with the MTR, and the difference was statistically significant, which is consistent with the results of research conducted in Boston, United States [8, 17]. The results of the multivariable analysis confirm this view. The probability of not completing PEP was higher when using TDF/FTC + DTG as the PEP regimen than when using BIC/FTC/TAF (aOR; 7.02, 95% CI: 1.82–46.29). The completion rate of BIC/FTC/TAF in this study was similar to that of a study conducted at the Beijing You Unk Hospital [12]. Both studies showed that the 28-day completion rate of BIC/FTC/TAF as a PEP regimen is exceptionally high, with rates of 97.8% and 96.4%, surpassing those observed with other single-tablet regimens [8, 11, 15, 24]. This high completion rate holds significant importance in PEP, given that non-completion may potentially correlate with subsequent HIV seroconversion [25]. A possible explanation for the high completion rate is that many studies have confirmed its safety and tolerance as an antiretroviral regimen [26–28], and it is currently the pill size is the smallest STR available in China.

In our study, one participant who used TDF/FTC + DTG stopped PEP because of intolerance (1.1%). The results indicated that both regimens exhibited good safety and tolerance.

This study has some limitations. First, it was a non-randomized study; the choice of the regimen was based on the preferences of the participants, and selection bias is likely. We used a multivariable logistic regression model to control for the influence of confounding factors as much as possible; however, the results may be biased in terms of safety reporting. Second, we did not monitor blood drug concentrations, which can reflect adherence more accurately. Third, our study included only sexual exposure. Finally, this was a single-center study, and thus generalizability of the results may be limited. Diverse geographical and social factors are very important to evaluate the universality of this research result; therefore, further multicenter prospective research studies are needed to confirm our findings in a more diverse study population.

## Conclusion

Our research shows that BIC/FTC/TAF, as an STR regimen for PEP, has a high completion rate, high adherence, good safety, and tolerance at 28 days; therefore, it can be used as the first choice for PEP.

## Data Availability

No datasets were generated or analysed during the current study.

## Abbreviations

ADR: Adverse drug reaction
BIC/FTC/TAF: Bictegravir, emtricitabine, and tenofovir alafenamide
MTR: Multi-tablet regimen
PEP: Post-exposure prophylaxis
STR: Single-tablet regimen
TDF/FTC+DTG: Tenofovir disoproxil fumarate, emtricitabine, and dolutegravir
